# Detection and Characterization of Enteropathogenic and Shiga Toxin-Producing *Escherichia coli* Strains in *Rattus* spp. from Buenos Aires

**DOI:** 10.3389/fmicb.2018.00199

**Published:** 2018-02-14

**Authors:** Ximena Blanco Crivelli, María P. Bonino, Paula Von Wernich Castillo, Armando Navarro, Osvaldo Degregorio, Adriana Bentancor

**Affiliations:** ^1^Microbiología, Facultad de Ciencias Veterinarias, Universidad de Buenos Aires, Buenos Aires, Argentina; ^2^Departamento de Salud Pública, Facultad de Medicina, Universidad Nacional de México, Mexico City, Mexico; ^3^Salud Pública, Facultad de Ciencias Veterinarias, Universidad de Buenos Aires, Buenos Aires, Argentina

**Keywords:** *Rattus*, synanthropic, EPEC, STEC, childhood diarrhea

## Abstract

Enteropathogenic *Escherichia coli* (EPEC) and Shiga toxin-producing *E. coli* (STEC) are pathovars of *E. coli* that impact human health by causing childhood diseases. In this work, 118 synanthropic rodents of the genus *Rattus* from Buenos Aires, Argentina were evaluated as EPEC and STEC carriers. Rectal swab samples from captured animals were evaluated by conventional PCR to detect the presence of the *eae*, *stx*1, *stx*2, and *rfb*O157 genes. Twenty-one isolates were obtained (17 EPEC isolates from seven animals and four STEC isolates from the same animal). All EPEC isolates tested negative for the presence of the *bfpA* gene. One EPEC isolate carried the *iha* gene, and five EPEC isolates carried the *tox*B gene. STEC isolates exhibited two different virulence profiles: *stx*1a/*stx*2a/*stx*2c/*stx*2d/*saa*/*ehx*A/*sub*A (3/4) and *stx*1a/*stx*2a/*saa*/*ehx*A/*sub*A (1/4). EPEC isolate serotypes included O109:H46 (7), O71:H40 (4), O71:NM (2), O138:H40 (1), O108:H21 (1), O88:H25 (1), and O76:NM (1), and STEC isolates belonged to the O108:H11 (4) serotype. Antimicrobial susceptibility testing was carried out, and resistance to tetracycline was observed in one EPEC strain. Our results demonstrate that *Rattus* spp. may act as carriers of EPEC and STEC strains and may be involved in the epidemiology of diarrheal disease in infancy.

## Introduction

*Escherichia coli* is a microorganism belonging to the *Enterobacteriaceae* family, whose habitat is the intestines of various animal species including humans. Non-pathogenic *E. coli* can become pathogenic following the acquisition of mobile genetic elements by horizontal transfer and interactions with bacteriophages or transposons that interface between *E. coli* and other bacterial species (Kaper et al., 2004; Williams et al., 2010). Non-pathogenic and pathogenic *E. coli* can be differentiated using molecular methods (Nataro and Kaper, 1998) and *in vitro* assays in cell lines (Konowalchuk et al., 1977).

Infections resulting from pathogenic *E. coli* may be limited to mucosal surfaces or spread throughout the body. Three clinical syndromes can result from infection with different *E. coli* pathovars: diarrheagenic disease, urinary tract infections, and sepsis/meningitis (Nataro and Kaper, 1998). Some *E. coli* pathovars that produce diarrheagenic disease cause histological lesions known as attaching and effacing (A/E) lesions in the gut mucosa of human and animal hosts (Nataro and Kaper, 1998). The formation of A/E lesions involves both plasmid and chromosomal genes that are encoded in a pathogenicity island known as the locus for enterocyte effacement (LEE) (McDaniel et al., 1995). The *eae* gene is present in the LEE and encodes intimin, an adhesin that mediates an intimate connection between the bacterium and the enterocyte, causing localized destruction of microvilli and the formation of an actin-rich pedestal-like structure on the apical cell membrane (Wales et al., 2005).

Enteropathogenic *E. coli* (EPEC) produce A/E lesions. EPEC strains have been identified as one of the causes of childhood diarrhea, which is responsible for 1000s of deaths worldwide (Ochoa et al., 2008). Cases of diarrhea caused by EPEC vary from subclinical to fatal infections (Torres et al., 2010). Children with clinical diarrhea caused by EPEC usually fail to respond to oral rehydration therapy, suffer from cow milk intolerance, and develop persistent diarrhea requiring hospitalization (Fagundes-Neto and Scaletsky, 2000). EPEC strains are classified into two groups, typical (tEPEC) and atypical (aEPEC), according to the presence or absence of the EPEC adherence factor plasmid (pEAF). This plasmid includes the *bfp* gene, which encodes the bundle-forming pilus necessary for localized adhesion in cell culture (Kaper et al., 2004). tEPEC strains have been isolated from humans, and aEPEC strains have been found in various animal species as well as humans (Gomes and González Pedrajo, 2010).

Shiga toxin-producing *E. coli* (STEC) is another pathovar that has an impact on childhood health. STEC is mainly characterized by the production of Shiga toxin (Stx), which binds to its receptor globotriaosylceramide (Gb3), disrupts protein synthesis, and kills epithelial or endothelial cells. Some STEC strains also carry the *eae* gene. The pathogenesis of STEC involves different presentations, from asymptomatic to mild or severe diarrhea, as well as hemolytic uremic syndrome (HUS), which may cause death. Ruminants, in particular cattle, have been identified as the main reservoir of these bacteria (Karmali, 1989). In addition, several animal species have been identified as hosts to this pathogen (Beutin et al., 1993; Leotta et al., 2006; Bentancor et al., 2007).

Furthermore, the presence of hybrid *E. coli* strains has been recorded in various parts of the world. In May 2011, an enteroaggregative *E. coli* O104:H4 transduced by a Stx2a-converting phage (EAEC/STEC) led to a food-borne outbreak in Germany with 3816 cases reported (including 54 deaths); 845 of those cases led to HUS (Frank et al., 2011). Similarly, the presence of EAEC/STEC hybrid strains associated with HUS and diarrhea has been recorded in Argentina since 2005 and has been associated with the O59:NM [H19] and ONT:H4 serogroups (Rivas, 2016). Nyholm et al. (2015) analyzed the genomes of three enterotoxigenic/STEC *E. coli* hybrid strains and concluded that virulence genes can co-exist in strains originating from different phylogenetic lineages. Considering the emergence of hybrid strains or heteropathogenic *E. coli*, the current genetic classification of strains into classical pathotypes is limited. Detection of hybrid pathovars is important because they may have a significant impact on public health.

The presence of EPEC and STEC strains in synanthropic animals, especially those which belong to the genus *Rattus*, is important because of the direct or indirect contact they may have with humans and household animals. The aim of this work was to evaluate synanthropic rodents of the *Rattus* genus from Buenos Aires, Argentina as carriers of EPEC and STEC strains and to assess the virulence profiles of these strains. Given the impact of diarrhea on children in Argentina, this study is of great importance to the epidemiology of transmissible diseases, as it provides health intervention criteria.

## Materials and Methods

### Samples

We conducted a cross-sectional epidemiological study to determine the presence of EPEC and STEC strains in rodents of the *Rattus* genus in Buenos Aires. Convenience non-probability sampling was carried out at accessible points in Buenos Aires, including three case animals that were temporally and spatially related to a HUS outbreak according to the National Health Surveillance System (restricted up to 50 m of HUS address within the week of the outbreak). The target population corresponded to *Rattus* rodents obtained in Buenos Aires; thus, all rodents belonging to the *Rattus* genus that were captured in urbanized areas of the city of Buenos Aires were included, while all non-*Rattus* rodents and non-rodent animals captured were excluded.

**FIGURE 1:**
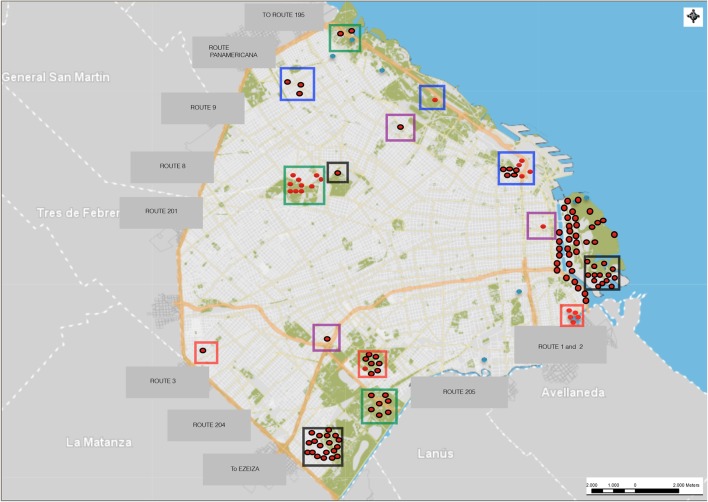
Sites of convenience sampling and trapping of *Rattus* spp. in Buenos Aires. Red dots: *Rattus rattus* captured; red dots with black outlines: *Rattus norvegicus* captured; light blue dots: sites with traps without capture; red squares: HUS outbreak sites; purple squares: residential sites; black squares: shantytowns; green squares: parkland sites; blue squares: railways; areas without squares: riverside.

Rats were captured using live traps (2620 cage traps) set in different areas of Buenos Aires (**Table 1**). The traps remained active for four consecutive nights, and they were checked each morning, resulting in a total of 118 rats captured (**Figure 1**).

**Table 1:** Locations and species of animals captured.

Capture area	*R. rattus*	*R. norvegicus*
Railways	5	8
Shantytowns		34
HUS outbreak sites	6	8
Parkland sites	10	9
Residential sites	1	2
Riverside		35
Total	22	96


Each rat was euthanized in accordance with the recommendations of the American Veterinary Medical Association guidelines for the euthanasia of animals (Underwood et al., 2013). We used a protocol that was approved by the Institutional Committee on Animal Care and Use of Experimental Animals (CICUAL; No. 2010726) of the Universidad de Buenos Aires, Facultad de Ciencias Veterinarias. Then, each rat was weighed, measured, sexed, and its reproductive status was recorded. Subsequently, the rats were classified according to species. All animals were sampled via two rectal swabs.

### Detection and Isolation of EPEC and STEC Strains

Samples were used to inoculate 5 ml of tryptone soy broth (TSB) for pre-enrichment and incubated for 18 h at 37°C to detect EPEC and non-O157 STEC strains. The cultures were streaked onto MacConkey agar (MAC) and incubated overnight. Screening for the *eae* gene was carried out by PCR of cells from the confluence zone using primers reported by Blanco et al. (2004) with a previously described modification (Bentancor et al., 2007). The presence of *eae*-positive strains was verified by screening up to 50 CFU of positive samples from the confluence zone. For the detection of non-O157 STEC strains, screening of the confluence area for *stx1* and *stx2* genes was carried out by PCR using primers reported by Leotta et al. (2005). Suspected samples were evaluated for the presence of *stx*1-positive or s*tx*2-positive strains by screening up to 50 CFU.

Furthermore, for the detection of O157 STEC strains, samples were inoculated in 5 ml of tellurite cefixime TSB for enrichment and incubated for 6 h at 37°C. Inmunomagnetic separation (IMS) with Dynabeads^®^ anti-*E. coli* O157 was performed. Each Dynabeads–bacterial complex was streaked onto sorbitol MacConkey agar (SMAC) and onto *E. coli* O157:H7 chromogenic medium (Chrom ID) and incubated for 18 h at 37°C. Screening for *stx*1/*stx*2 and *rfb*O157 from the confluence area was performed by multiplex PCR (Leotta et al., 2005).

### Detection Limit of IMS in Rodent Stool Samples

In order to establish the detection limit of the IMS in rodent stool, *E. coli* EDL 933 (positive control strain) was suspended in saline solution at a McFarland turbidity of 0.5. Viable cell count was determined using 10-fold dilutions and culture in brain heart agar (BHA). In addition, a pool of STEC-negative (NS) rodent stool pre-cultures that had been recovered previously was assessed. A total of 100 μl of each EDL 933 dilution was added to 900 μl of NS culture. IMS was performed for each tube according to the recommendations of the supplier by adding 20 μl of immunomagnetic beads and subsequently seeding in SMAC. Finally, PCR was performed to detect *stx*1, *stx*2, and *rfb*O157 from confluent growth zone. This test was repeated three times.

### Biochemical Characterization and Identification of Additional Virulence Factors

Isolates were further characterized using biochemical tests (MacFaddin, 2003). Additional virulence factors such as *saa*, *ehx*A (Paton and Paton, 1998, 2002), the putative adhesion factor *tox*B (Toma et al., 2004), the iron regulatory gene *iha* (Schmidt et al., 2001), the cytolethal distending toxin *cdt*III (Clark et al., 2002), *cdt*VA, *cdt*VB (Janka et al., 2003), and subtilase (*sub*A) (Paton and Paton, 2005) were also detected by PCR according to methods reported in the above previous studies.

### Identification of *E. coli* Diarrheagenic Hybrid Pathovars

Other markers in *E. coli* diarrheagenic pathovars were evaluated by conventional PCR, including the *aai*C (Boisen et al., 2012) and *aag*R (Wieler et al., 2011) genes for enteroaggregative *E. coli* (EAEC), *elt* and *est*A (Itoh et al., 1992) for enterotoxigenic *E. coli* (ETEC), *daa*E (Vidal et al., 2005) for diffusely adherent *E. coli* (DAEC), and *inv*E (Itoh et al., 1992) for enteroinvasive *E. coli* (EIEC).

### Detection of *bfp*A-Positive EPEC Strains

Strains positive for *eae* and negative for *stx* were further analyzed to detect the *bfp*A gene. The primers used and the PCR conditions have been previously described (Gunzburg et al., 1995).

### Subtyping of *stx-*Positive STEC Strains

Strains positive for *stx* were further analyzed to detect subtypes of *stx*1 and *stx*2 by PCR. The primers used and the PCR conditions have been previously described (Scheutz et al., 2012).

### Production of Shiga Toxin by STEC Strains

The production of Stx1 and Stx2 was detected with an immunochromatographic test (Duopath Verotoxin test; Merck) (Park et al., 2003). The detection limit of this test is 25 ng/ml for Stx1 and 62.5 ng/ml for Stx2. Each isolate was inoculated in blood broth and simultaneously in 2 ml of Caye broth with Caye supplement, then incubated for 24 h at 37°C and 6 h at 37°C, respectively, following the recommendations of the supplier. Subsequently, cultures were treated with polymyxin B sulfate in a ratio of 9:1 (360 μl broth + 40 μl antibiotic) and incubated in a dry bath (Accu Block, Labnet) for 10 min at 37°C. An aliquot of the inoculum was transferred to the sample port, the test device was incubated at room temperature, and the result was interpreted after 10 min.

### Enterohemolysin Production

For *ehx*A-positive strains, we investigated the production of enterohemolysin on tryptose blood agar base supplemented with 10 mM CaCl_2_ and 5% defibrinated sheep blood (Beutin et al., 1989).

### Serotyping of Isolated Strains

Somatic antigen (O) and flagellar antigen (H) were examined according to a previously reported methodology (Orskov and Orskov, 1984). We used 187 rabbit sera against somatic antigens (O) and 53 rabbit sera against flagellar antigens (H) from the *E. coli* antigenic scheme. The rabbit sera were previously obtained using reference strains of *E. coli* according to a proposed method by Ewing (1986).

### Antimicrobial Susceptibility Testing

Isolated strains were examined for resistance to amoxicillin/clavulanic acid (30 μg), imipenem (10 μg), aztreonam (30 μg), gentamicin (10 μg), amikacin (30 μg), nalidixic acid (30 μg), ciprofloxacin (5 μg), nitrofurantoin (300 μg), and tetracycline (30 μg) using commercial monodisks (Oxoid) and tablets (Rosco) by the disk diffusion method in Müeller Hinton agar as recommended by the Clinical Laboratory and Standards Institute (CLSI). Strains were characterized as being susceptible, having reduced susceptibility, or being resistant (Clinical Laboratory and Standards Institute [CLSI], 2014).

### Statistical Analysis

The data obtained were analyzed using Fisher’s exact test (InfoStat 2016e).

## Results

Among the *Rattus* spp. captured, 96 were *R. norvegicus* (81%) and 22 were *R. rattus* (19%). The proportion of EPEC (*eae*-positive) carriers among *R. norvegicus* specimens was 3.12% (3/96). Most of these belonged to serotype O109:H46, followed by O108:H21 and O138:H21 (**Table 2**). Interestingly, multiple isolates belonging to serotype O109:H46 recovered from the same *R. norvegicus* specimen exhibited distinct characteristics: while one isolate was *eae*+/*iha*+, the others were *eae*+/*toxB*+. All *R. norvegicus* EPEC carriers were captured from shantytown sites (**Table 2**).

**Table 2:** Enteropathogenic and Shiga toxin-producing *Escherichia coli* isolated from *Rattus* spp.

	Serotype	Genetic markers	Total number of positive animals	Place of capture	Total number of strains isolated	Diarrheagenic category
*R. norvegicus*	O109:H46	*eae, toxB*	1^∗^	Shantytown	5	EPEC
	O109:H46	*eae, iha*	1^∗^	Shantytown	1	EPEC
	O108:H21	*eae*	1	Shantytown	1	EPEC
	O138:H40	*eae*	1	Shantytown	1	EPEC
*R. rattus*	O71:H40	*eae*	1^∗∗^	Parkland site	4	EPEC
	O71:NM	*eae*	1^∗∗^	Parkland site	2	EPEC
	O76:NM	*eae*	1	Parkland site	1	EPEC
	O88:H25	*eae*	1	Parkland site	1	EPEC
	O109:H46	*eae*	1	Parkland site	1	EPEC
	O108:H11	*stx*1^a^, *stx*2^b^, *saa*, *ehx*A, *sub*A	1	HUS outbreak	4	STEC


**^∗^Two strains isolated from the same animal, differing in genetic markers. ^∗∗^Two strains isolated from the same sample, differing in serotype but with the same genetic markers. ^*a*^stx1 subtype: stx1a (n = 4). ^*b*^sxt2 subtypes: stx2a (n = 1), stx2a/stx2c (n = 3). Genes rfbO157, bfp, cdtIII, cdtVA, cdtVB, aaiC, aagR, elt, est, daaE, and invE were not detected*.*

The proportion of EPEC carriers among *R. rattus* specimens was 18.18% (4/22). Two EPEC strains isolated from the same sample and belonging to the O71 serogroup belonged to different serotypes (O71:H40/NM) according to serological tests. All *R. rattus* EPEC carriers were captured from parkland sites (**Table 2**).

Based on these results, there was a statistically significant difference in the rate of EPEC strains carried by the two *Rattus* species (*p* = 0.04).

A total of 17 isolates were obtained from the seven EPEC-positive *Rattus* specimens (**Table 2**). All *eae*-positive isolates were negative for *stx*1, *stx*2, *saa*, *ehx*A, *cdt*III, *cdt*VA, *cdt*VB, and *sub*A. In addition, all *eae*-positive isolates were identified as *E. coli* and were classified as aEPEC (*bfp*A-negative). One EPEC isolate was *iha*-positive, and five EPEC isolates were *iha*-negative/*tox*B-positive. Resistance to tetracycline was observed in one EPEC strain.

Only one sample obtained from an *R. rattus* specimen captured during a HUS outbreak was found to be *stx*1/*stx*2-positive/*rfb*O157-negative/*eae*-negative. Four isolates were obtained from this animal (**Table 2**), all of which were identified as *E. coli* and classified as STEC. These strains exhibited the genetic profiles *stx*1a/*stx*2a/*stx2c/saa/ehxA/subA* (3/4) and *stx*1a/*stx*2a/*saa/ehxA/subA* (1/4). All STEC isolates produced Stx1 and Stx2 and exhibited an enterohemolytic phenotype.

The serotypes of the strains isolated in this study are shown in **Table 2**. Other markers for *E. coli* diarrheagenic pathovars were not detected in EPEC or STEC isolates, and there were no hybrids. The detection limit for IMS in rodent stool samples was established to be 12.6 × 10^2^ CFU/ml.

## Discussion

In this study, we evaluated the presence of EPEC and STEC strains in rodents of the genus *Rattus*. The sampling methodology (rectal swab) differed from that used by other researchers working with synanthropic rodents. While Nielsen et al. (2004) took fecal samples from the environment, Rahn et al. (1997) collected feces present in live catch traps for rodents, and Hancock et al. (1998) and Cižek et al. (1999) used feces from the gastrointestinal tracts of captured animals. However, in this study, rectal swabs were used for sampling, as Greenquist et al. (2005) used swabs of the rectoanal junction mucosa to identify the presence of STEC O157 and determine the epidemiological role of cattle as a reservoir. Moreover, the presence of STEC O157 in stool samples can be related to the transient microbiota in these samples.

In this work, we isolated EPEC strains, and our results were similar to those obtained by other authors (Heidemanns et al., 2009; Firth et al., 2014). Ten animals were suspected to be carriers of the pathogen, and we isolated EPEC strains from seven animals. A higher proportion of EPEC strains were found among *R. rattus* specimens; this result is similar to a previous finding by our group (Rumi et al., 2012). However, we also found EPEC strains in *R. norvegicus*, a finding which is similar to those obtained by Firth et al. (2014). All EPEC isolates were classified as aEPEC because they tested negative for the *bfp*A gene. Certain features may favor the prolonged intestinal colonization of aEPEC compared to other intestinal pathogens, allowing aEPEC strains to persist for a longer period of time in the intestine (Ochoa et al., 2008). Currently, it has been reported that the proportion of aEPEC strains has increased and is associated with childhood diarrhea, while the proportion of tEPEC strains is declining (Ochoa et al., 2008; Hu and Torres, 2015); this could also be a possible explanation for the high proportion of aEPEC strains among the isolates.

The differences in the carriage of EPEC between species in this study should not be considered due to geographic clustering because we carried out a convenience non-probability sampling. Thus, this finding should be interpreted cautiously and further studies should be performed in order to examine if the difference in carriage between the species is real.

EPEC O71:H40, which was isolated from *R. rattus* (*n* = 4), has previously been isolated from ruminants (Horcajo et al., 2012) and from patients with diarrhea or other gastrointestinal disturbances (Hien et al., 2008; Horcajo et al., 2012). Moreover, an EPEC isolate from an *R. rattus* specimen presented the serotype O88:H25, which has a high incidence in Brazil without being associated with cases of diarrhea in humans (Pedroso et al., 1993). This has also been identified as a new serotype in Mexico, where it was isolated from children under 5 years of age with acute diarrhea and bloody diarrhea (Navarro and Estrada-García, 2010).

To our knowledge, there are no previous records of serotypes O138:H40, O109:H46, O108:H21, and O76:NM among EPEC strains, indicating that this study is the first to identify aEPEC isolates of these serotypes.

Inmunomagnetic separation appears to be an efficient method for the detection of STEC O157, as the detection limit in rodent stool samples was 12.6 × 10^2^ CFU/ml. This value is lower than that obtained by Bentancor et al. (2007) for dog swabs (6.6 × 10^3^ CFU/ml). However, the possibility of competition by the resident microbiota of the gastrointestinal tracts of rodents cannot be ruled out. It has been established that cross-reactions with antigenically similar microorganisms such as *Escherichia hermannii*, *Salmonella* spp., and *Proteus* spp. may occur in IMS. Non-specific binding with microorganisms such as *Pseudomonas* spp. and *Serratia liquefaciens* may also occur (Dynal Biotech A S.A.). Although the microbiotas of synanthropic *Rattus* spp. were not analyzed, they could include a high proportion of one of these microorganisms.

Screening for STEC O157 was negative, even though a selective enrichment protocol was used. The absence of STEC O157 in rat samples is in agreement with results obtained by other authors (Hancock et al., 1998; Rahn et al., 1997; Nielsen et al., 2004). However, Cižek et al. (1999) found STEC O157 in *R. norvegicus*. The low rate of detection of this pathogen in *Rattus* spp. may be due to the fact that it can be excreted quickly or sporadically by these animals, and we should not rule out the possibility of the existence of carriers with levels of the pathogen below the detection limit of the diagnostic test.

The screening for non-O157 STEC strains identified four isolates from a *R. rattus* specimen. The presence of non-O157 STEC in *Rattus* spp. coincides with the results obtained by Nielsen et al. (2004), who isolated STEC O136:H12 (*stx*1-positive) in *R. norvegicus*. However, there is no history of STEC isolates in *R. rattus*, except for in our previous study involving a HUS outbreak (Bentancor et al., 2009; Blanco Crivelli et al., 2012).

Shiga toxin-producing *E. coli* isolates carried *sub*A, a gene that codes for subunit A of SubAB. The presence of this cytotoxin in non-O157 STEC strains was previously recorded (Paton et al., 2004). This toxin is 10–100 times more potent than Stx in Vero cells (Noda et al., 1987; Fujii et al., 2003) and may play a role in the pathogenesis of HUS (Irino et al., 2010).

The enterohemolytic phenotype was observed in all STEC isolates. This is important because enterohemolysis is associated with diarrheal disease in humans (Beutin et al., 1989). Moreover, all the STEC isolates obtained in this work corresponded to the serotype O108:H11 and the profile *stx*1/*stx*2/*saa*/*ehx*A/*sub*A. Toxin production was verified by immunochromatography, which has a sensitivity of 100% and results in no false positives when testing individual fecal cultures (Park et al., 2003).

The STEC-positive *Rattus* specimen that was captured was related to a HUS outbreak according to the National Health Surveillance System. In this case, no STEC strains were isolated from affected children, so it is not possible to establish a connection with our isolated strains.

Only one EPEC isolate showed resistance to tetracycline. This is expected among wild-type strains obtained from synanthropic animals, suggesting that the isolated strains originated among the microbiota of *Rattus* spp. and are not derived from other sources, such as human waste, which may could have been ingested by these animals.

The typical intestinal inhabitants of rodents are not well-known, so it is possible that the intestines of these animals constitute a niche that favors variability among the strains (Heidemanns et al., 2009). Our results demonstrated that *Rattus* spp. can be carriers of EPEC and STEC strains. Moreover, we found a significant association between the *Rattus* species and the presence of EPEC. In Argentina, studies of the presence of EPEC in urban, domestic, and synanthropic animals are limited. There are few epidemiological studies of EPEC in dogs and cats, and to our knowledge, this is the first report describing synanthropic carriers of EPEC in urban areas of Argentina. Synanthropic rodents from the *Rattus* genus could play a role in the epidemiology of EPEC. It is therefore imperative to perform continuous monitoring and identification of carriers and risk factors for EPEC, STEC, and other *E. coli* pathovars of significance for public health.

## Author Contributions

Conceived and designed the experiments: AB and XB. Performed the experiments: XB, MB, PVWC, AN, and AB. Analyzed the data and wrote the paper: XB, OD, and AB.

## Conflict of Interest Statement

The authors declare that the research was conducted in the absence of any commercial or financial relationships that could be construed as a potential conflict of interest.
